# NMR Spectroscopy of supramolecular chemistry on protein surfaces

**DOI:** 10.3762/bjoc.16.203

**Published:** 2020-10-09

**Authors:** Peter Bayer, Anja Matena, Christine Beuck

**Affiliations:** 1 Structural and Medicinal Biochemistry, University of Duisburg-Essen, Universitätsstr. 1-5, 45141 Essen, Germany

**Keywords:** molecular recognition, NMR, protein ligand interaction, protein surfaces, supramolecular chemistry

## Abstract

As one of the few analytical methods that offer atomic resolution, NMR spectroscopy is a valuable tool to study the interaction of proteins with their interaction partners, both biomolecules and synthetic ligands. In recent years, the focus in chemistry has kept expanding from targeting small binding pockets in proteins to recognizing patches on protein surfaces, mostly via supramolecular chemistry, with the goal to modulate protein–protein interactions. Here we present NMR methods that have been applied to characterize these molecular interactions and discuss the challenges of this endeavor.

## Introduction

In recent years, the focus of biochemical research and drug development has shifted from the inhibition of single enzymes to targeting protein-protein interactions [1–2], which play key roles in cellular function and dysfunction [3–4]. Enzymes usually bind their substrates in deep pockets with specific shapes and chemical properties. In contrast, the interaction between two proteins often involves docking of larger patches on the protein surface, which are complementary in shape and charge. The specific recognition of these patches by synthetic molecules poses challenges because these areas on the protein surface are shallow and a similar composition of hydrophilic and charged residues is often found on multiple proteins. To specifically address such an area on a protein without risking non-specific binding to many others, the topology of the surface - including shape, surface accessibility as well as distance of targeted residues and their dynamics - must be taken into account. Supramolecular chemistry is ideally suited to address these challenges because it merges the knowledge of non-covalent molecular recognition with the possibility to combine multiple recognition units into one molecule for improved selectivity and affinity. Examples for supramolecular ligands designed to recognize protein surfaces include tweezers [5–18], calixarenes [19–40], guanidiniocarbonylpyrrole (GCP) ligands [41–46], cucurbiturils [47–51], porphyrins [52–60], metal complexes [61–63], foldamers [64–67], nanoparticles [34,68], imprinted polymers [69–72], and dendrimers [73–74].

**Figure 1:**
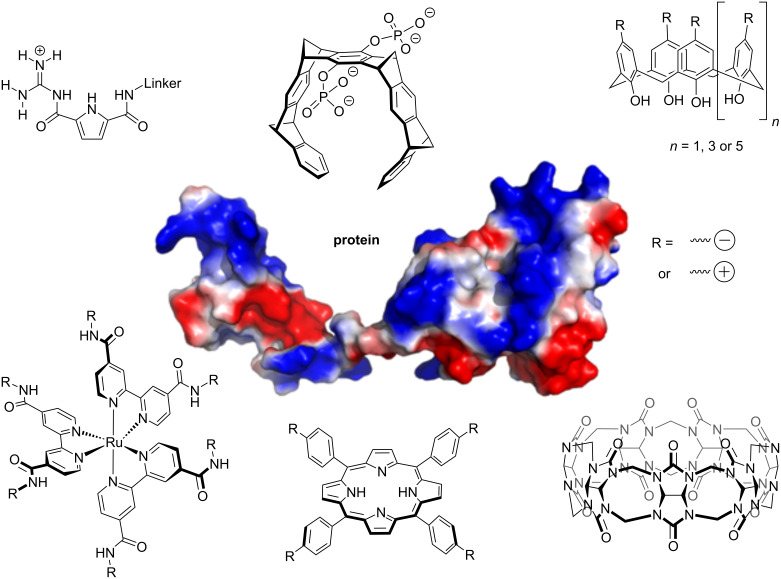
Ligands targeting charged areas on protein surfaces discussed in this review. The protein shown as example is *h*Pin1 (pdb 1NMV, [75]). The guanidiniocarbonylpyrrole motif (GCP, top left) recognizes the carboxylate groups of aspartate or glutamate residues by forming an extended H-bonding network [41–46]. Supramolecular tweezers (top center) thread lysine or arginine side chains into their aromatic cavities [5–18]. Calixarenes (top right, [19–40]), Ru^II^(bpy)_3_ complexes (bottom left, [61–63]) and porphyrins (bottom center, [52–60]) can be functionalized with either multiple acidic or basic groups to target charged areas of either polarity on a protein surface. Cucurbiturils (bottom right, [47–51]) recognize methylated lysines and arginines by binding their methylated head groups inside the macrocycle.

In this review, we focus mostly on ligands that recognize either positively or negatively charged patches on a protein surface, discussing the molecules shown in Figure 1 in greater detail. Ligands designed to recognize positively charged regions, containing lysine (Lys) and arginine (Arg) residues, on a protein include supramolecular tweezers [5–18] as well as sulfonato- and phosphonato-calixarenes [19–38]. These ligands contain negatively charged functionalities interacting with the positively charged head groups of Lys and Arg, often combined with a moiety that cradles the hydrophobic portion of the aliphatic side chain.

Supramolecular tweezers consist of alternating benzene and norbornadiene units and thread the side chain of Lys or Arg into their aromatic cavity. In addition, an ion pair interaction is formed between the charged head group of the encapsulated amino acid and one of the phosphate moieties attached to the tweezer’s central benzene ring [5]. Supramolecular tweezers have not only proven to be interesting tools to modulate protein–protein interactions [6–7], reverse amyloid fibril formation [8–12], and inhibit enzyme function [13] in vitro, they also exhibit interesting effects in vivo like the reversal of Alzheimer plaques in mice [14], tumor inhibition [15], and reduction of HIV infectivity [16], all while showing almost no toxic side effects [14,17].

In calixarenes with negatively charged sulfonate [20–22,24–25,27–32,34,36–37] or phosphonate [23,33,35] substituents, the aliphatic side chain of the amino acid lines the aromatic bowl-like structure of the calixarene, while the positively charged end group being situated between the sulfonate or phosphonate groups. While sulfonato-calix[4]arenes can bind unmodified Lys and Arg residues, their strength lies in the recognition and even tighter binding of methylated lysines [29]. Their binding affinity increases 70-fold from unmethylated over mono- and di- to trimethylated lysine, as every methyl group adds more hydrophobic interactions with the aromatic side walls of the bowl-shaped calixarene core. A similar, but even more pronounced trend, is observed for the pumpkin-shaped macrocycle cucurbit[7]uril, favoring the trimethylated over unmethylated lysine by a factor of 3500 [49]. Methylated lysines and arginines occur as posttranslational modifications in proteins and are particularly important in histones, where they contribute to the regulation of chromatin structure and thus gene expression. The calixarenes can successfully bind to methylated histone tails and thus inhibit the binding of epigenetic reader proteins [30–32]. Negatively charged calixarene ligands have further been used to inhibit enzymatic activity [33–35] and amyloid fibril formation [36–38].

Calix[4]arenes with positively charged substituents like guanidinium groups lining the top of their bowl-shaped core have been used to design custom plugs for the negatively charged central cavity of tetrameric proteins, exploiting the four-fold symmetry of the ligand [39–40]. In the case of tetrameric voltage-dependent potassium channels, binding of the calixarene to the central pore of the channel results in reversible inhibition of potassium ion transport [39]. Another guanidinium-calixarene variant was designed to hold together the four tetramer subunits of a p53-R337H tumor suppressor mutant and thus restore tetramer stability [40].

Further examples for ligands targeting negatively charged patches on proteins include guanidinocarbonylpyrrole (GCP)-based ligands [41]. GCP mimics the natural amino acid arginine binding to the carboxylate side chains of aspartate (Asp) and glutamate (Glu). The GCP unit provides an extended hydrogen bonding/salt bridge interface, which leads to better binding compared to its natural counterpart. The combination of two or more GCP moieties with variable linkers enables the construction of multivalent ligands geared towards a specific spot on protein surface, which by design can lead to either stabilization [42–45] or inhibition [46] of a given protein–protein interaction. The inhibition of the Survivin – Histone H3 interaction by a GCP ligand has been shown to inhibit tumor proliferation in cell culture [46].

Porphyrins, either with or without a complexed metal ion in the center, have been used as planar hydrophobic scaffolds where either negatively charged carboxylate or positively charged amino substituents can be attached [52–60]. Several porphyrin ligands show tight (nM) binding and can cause unfolding of the targeted protein [52–55]. However, other porphyrin ligands were used to recognize protein surfaces in a native state [56–57], promoted protein multimerization [60], and have shown antiviral properties [58].

Metal complexes, such as Ru^II^(bpy)_3_ complexes, have been designed as protein surface mimetics to target charged areas on protein surfaces [61–63]. The ligand sphere of the metal can be modified to recognize either positively or negatively charged patches, depending on the functional groups present.

X-ray crystallography is a useful tool to obtain structural information about the binding modes of supramolecular ligands on proteins (reviewed in [76]). However, the crystallization of proteins with supramolecular ligands can be very challenging due to their often moderate (µM) binding affinities. If crystal contacts between proteins are favored, this can lead to exclusion of the ligand from the crystal. On the contrary, ligands binding tightly to the protein surface can in some cases compete with crystal packing and thus inhibit crystallization. Crystallization seems to succeed best for ligands that can adopt a defined conformation upon binding without flexible loops protruding from the protein surface. Even in these cases, the ligand is preferably found at binding sites that are facing solvent channels but at the same time enable stabilization of the ligand by additional crystal contacts to neighboring proteins [6,18,20–22,26–28]. For some supramolecular ligands, like sulfonatocalix[8]arene or phosphonatocalix[6]arene, the tendency to bridge two protein interfaces that do not usually interact in solution goes so far that they have been used to serve as scaffolds for the assembly of protein arrays and crystallization aids [23–28]. In addition, a terbium^III^ complex termed crystallophore has been developed as a crystallographic nucleation and phasing tool for protein crystallography that can also easily be detected by its luminescence [77–79]. Overall, while crystal structures provide fantastic insight into molecular interactions of such supramolecular ligands, they do not necessarily reflect the binding situation in solution. Furthermore, to our knowledge no protein co-crystal structures with multivalent ligands, like multi-armed GCP ligands with flexible scaffolds or nanoparticles, have been published yet.

NMR spectroscopy is the ideal method to gain structural information and identify residues involved in ligand binding because it delivers single-residue resolution. It is also suitable to characterize the binding of ligands with moderate dissociation constants (*K*_D_) in the µM (up to mM) range, where the *K*_D_s for many first-generation supramolecular ligands lie. In the case of multiple ligand binding sites on one protein surface, NMR allows to identify the involved areas and provides an idea about the ligand:protein stoichiometry, which can be difficult to obtain from classical biochemical binding assays, when multiple binding sites with potentially different *K*_D_s are involved. Both chemical shift perturbation and changes in signal intensity, mostly of the amide resonances, are used to map the binding site. Here we discuss protein-based solution NMR techniques including classic ^1^H,^15^N-HSQC spectra, TROSY variants for large proteins, fast acquisition techniques and specific isotope labeling strategies, as well as the use of ^13^C-edited spectra and side chain specific spectra for lysine and arginine residues for this purpose.

In this review, we focus on the use of protein-based NMR techniques to identify the binding sites of supramolecular ligands, particularly those recognizing charged patches, on protein surfaces. Table 1 presents a summary of the discussed NMR studies. We begin with a look at simple 1D proton spectra to study ligand binding to single amino acids or small peptides to introduce the basic principles and also briefly discuss ligand-detected NMR screening methods.

**Table 1:** Summary of NMR studies on supramolecular ligands binding to protein surfaces.

Supramolecular ligand	Protein name, function	Main interaction site(s)	Ligand function	Ref.

molecular tweezer (CLR01)	p97 N-domain, cofactor binding domain of AAA ATPase	6 patches with Lys & Arg: K20, K63/K60, K148/R113, R86/R144/R155, R159/K164, K190	inhibition of protein–protein interaction with cofactor UBXD1	[7]
molecular tweezer (CLR01)	hPin1-WW domain, substrate binding domain of peptidyl prolyl *cis-trans* isomerase	Lys & Arg, sterically accessible with basic residue in vicinity; preferred site R17, no binding to R36	NMR methodology study	[80]
molecular tweezer (CLR01)	tau, microtubule-binding protein, amyloid-forming	multiple Lys residues	inhibition of Tau fibril formation	[81]
molecular tweezer (CLR01)	ubiquitin, protein quality control	Arg-rich C-terminus (most sterically accessible) & Lys 48	binding model study	[82]
meso-tetrakis(4-sulfonatophenyl)- porphyrin (TPPS)	ubiquitin, protein quality control	hydrophobic triad (L8, I44, V70) and surrounding cationic residues	binding model study	[82]
pyrene tetrasulfonic acid (4PSA)	ubiquitin, protein quality control	most (9 out of 12) cationic residues	binding model study	[82]
sulfonato-calix[4]arene (SCLX4)	ubiquitin, protein quality control	Arg-rich C-terminus (most sterically accessible)	binding model study	[82]
sulfonato-calix[4]arene (SCLX4)	cytochrome c, electron carrier protein	large Lys-containing patches around K87 & K4; at least 2 sites per protein	binding model study; protein camouflage	[20]
phosphonato-calix[6]arene (PCLX6)	cytochrome c, electron carrier protein	N- and C-terminus with K4, K11, K100; at least 2 sites per protein	ligand-induced protein dimers in solution, ligand-induced protein assembly in crystal	[23]
sulfonato-calix[8]arene (SCLX8)	cytochrome c, electron carrier protein	large Lys-containing patches around K4; K86/K87, K100; at least 2 sites per protein	ligand-induced protein tetramers in solution, dissociation of tetramers with excess (>2×) ligand; ligand-induced protein assembly in crystal	[24]
*p*-phosphonatomethyl-calix[4]arene (PMCLX4)	cytochrome c, electron carrier protein	large Lys-containing patches, largest shifts around K86/K87, line broadening around K4, K11; at least 2 sites per protein	binding model study; ligand-induced protein assembly	[26]
sulfonato-calix[4]arene (SCLX4)	*Penicillium chrysogenum* antifungal protein (PAF), antifungal protein	K30	binding model study; ligand-induced protein assembly	[25]
sulfonato-calix[6]arene (SCLX6)	*Penicillium chrysogenum* antifungal protein (PAF), antifungal protein	K30 and weaker binding to K6, K42; strong line broadening	binding model study; ligand-induced protein assembly	[25]
sulfonato-calix[8]arene (SCLX8)	*Penicillium chrysogenum* antifungal protein (PAF), antifungal protein	K30, strong line broadening	binding model study; ligand-induced protein assembly	[25]
bromo-sulfonato-calix[4]arene (Br.SCLX)	cytochrome c, electron carrier protein	large Lys-containing patches, strongest shifts at K86; line broadening	binding model study	[27]
phenyl-sulfonato-calix[4]arene (Ph.SCLX)	cytochrome c, electron carrier protein	large Lys-containing patches, strongest shifts around K4/K5 and K86; severe line broadening	binding model study	[27]
PEGylated sulfonato-calix[4]arenes (SCLX4-PEG_1_ and SCLX4-PEG_2_)	cytochrome c, electron carrier protein	large Lys-containing patches, tight binding to K4, weaker binding to K86	binding model study	[28]
sulfonato-calix[4]arenes with additional aromatic substituent	K9-trimethylated histone 3 peptide, chromosome organization	trimethyl-Lys (K9Me_3_)	inhibition of interaction with plant homeodomain (PHD) of chromodomain helicase DNA-binding protein 4 (CHD4)	[31]
sulfonato-calix[4]arenes with additional aromatic substituent	K4-trimethylated methylated histone 3 peptide, chromosome organization	trimethyl-Lys (K4Me_3_), trimethyl-Lys (K9Me_3_)	up to 10× selectivity towards H3K4Me3 over H3K9Me3, inhibition of interaction with ING2 plant homeodomain (PHD)	[32]
guanidiniomethyl-calix[4]arene with 2 hydrophobic loops at the narrow rim	tetramerization domain (TD) of p53, transcription factor, tumor suppressor	hydrophobic clefts between two of the monomers each, at each side of the tetramer	recovering the tetramer integrity and stability of p53-R337H mutant protein	[40]
cucurbit[7]uril	*R. solanacearum* lectin, carbohydrate-binding protein	dimethylated lysine (KMe_2_), sterically accessible	KMe_2_ recognition, protein assembly	[50–51]
bivalent guanidinocarbonyl-pyrrole (GCP) ligand	survivin (residues 1-120), anti-apoptotic protein, part of chromosomal passenger complex	Glu/Asp-rich histone H3 binding site	inhibition of protein–protein interaction with histone 3	[46]
porphyrins with carboxylate substituents (Coproporphyrin I and 5,10,15,20-Tetrakis(3,5-dicarboxylato- phenyl)-porphyrin)	cytochrome c, electron carrier protein	dynamic ensemble including patches with varying hydrophobicity and positive electrostatic charge, overall 1:1 stoichiometry	binding model study	[59]
Ru^II^(bpy)_3_ complexes with carboxylate substituents	cytochrome c, electron carrier protein	Cyt c peroxidase binding site (ring-shaped positively charged patch)	inhibition of protein–protein interaction with Cyt c peroxidase	[63]

## Review

### One-dimensional ^1^H NMR spectra and basic principles to study ligand binding by NMR

Simple one-dimensional proton (^1^H) NMR spectra have been used to monitor the interactions between smaller molecules for a long time. The advantages comprise the use of non-isotope-labeled molecules and the fast acquisition time. Furthermore, both components - supramolecular host and guest - can in principle be monitored while titrating the other binding partner. However, for larger supramolecular hosts that encapsulate a large part of their (usually smaller) guest, it is advantageous to monitor the guest’s resonances. Monitoring binding with this simple spectrum requires at least one signal that does not overlap with signals from the binding partner. Therefore, this approach is only suitable to study binding of hosts to small guest molecules like amino acids and short peptides. Both a shifting of the signals (chemical shift perturbation) and line broadening are indicative of binding. The chemical shift perturbation is a direct measure of the fraction bound and can be used directly to determine the dissociation constant (*K*_D_), provided sufficient saturation can be reached [83].

Line broadening occurs due to chemical exchange, that is the interconversion of at least two species in an equilibrium, e.g., the free and bound form [84]. The extent of line broadening depends on the kinetics of the exchange between the two forms. If the exchange is slow relative to the NMR time scale, which roughly comprises a millisecond time frame, two separate sets of signals for both components are observed, where the signal integrals reflect the relative amounts of both states. If the exchange is fast relative to the NMR time scale, an averaged signal is observed, where the chemical shift, and thus the position of the signal, reflects the relative amounts of both species. If the exchange rate lies between these two extremes, line broadening and eventually coalescence of the signals from both species occurs. Often, the broadening of the signal results in a disappearing of the signal when the peak height drops below the noise level of the spectrum. In the case of intermediate-to-fast exchange, where most interactions with biomolecules with *K*_D_s in the µM range fall, the line shape and signal intensity do not directly correlate with fraction bound. It is possible to extract the kinetic interconversion rates from a full line shape analysis. The *K*_D_ can then be calculated from the ratio of the on and off rate (*K*_D_ = *k*_off_/*k*_on_). However, this requires knowledge of the chemical shifts and relaxation rates for both, the free and bound components in pure form, which are challenging to obtain for complexes with only moderate affinity.

For example, the binding of a single GCP unit to the carboxylates of different *N*-acetylated amino acids was monitored by tracking the amide HN and the binding constants were obtained from the resulting binding curves [41]. The binding constants of the amino acids tested cover a ≈ 5-fold range. The differences due to the amino acid side chains were investigated by molecular modeling. Ac-Phe shows the best binding (*K*_D_ = 0.6 mM) due to π-stacking between the phenyl side chain of the amino acid and the acylguanidinium unit of the GCP. Ac-Lys exhibits the least interaction (*K*_D_ = 3 mM) due to electrostatic repulsion of the positively charged Lys side chain with the also positively charged GCP unit.

**Figure 2:**
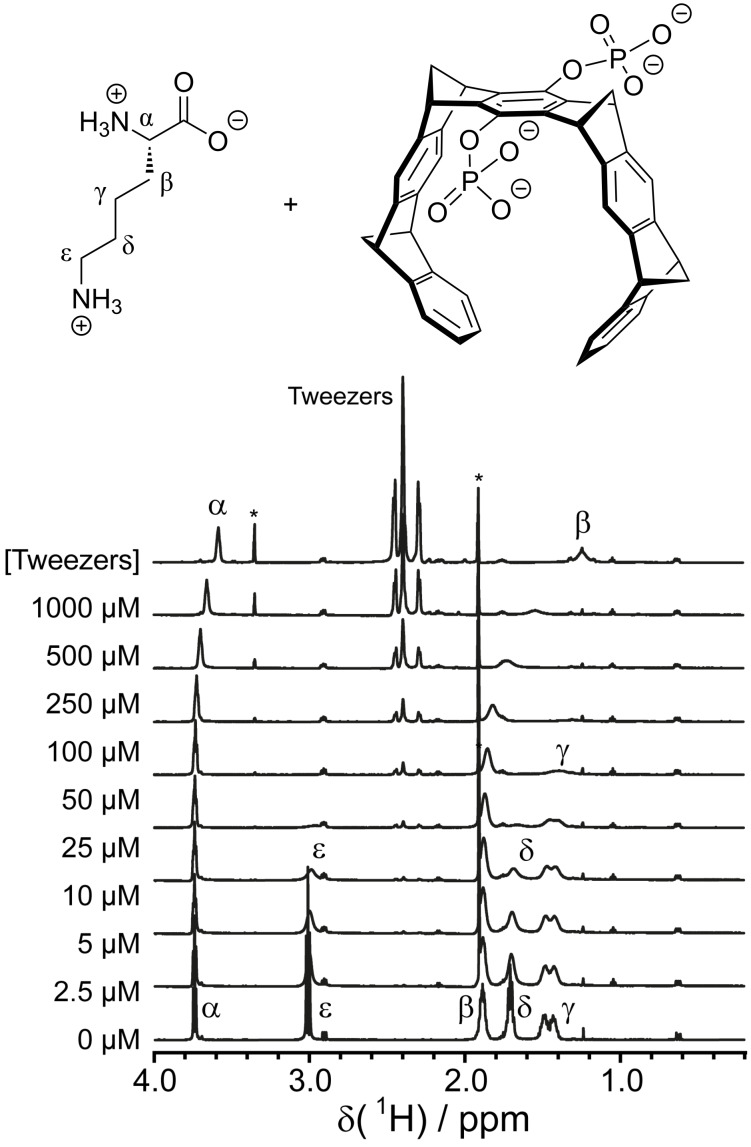
^1^H NMR titration of lysine with tweezers. All signals show chemical shift perturbations and different degrees of line broadening. The protons that are located right inside the tweezer cavity upon binding experience the largest effects. The asterisk marks impurities in the tweezers stock solution. Reprinted (reproduced) with permission from [80], copyright (2017) Wiley-VCH Verlag GmbH & Co. KGaA.

For supramolecular tweezers that bind to lysine or arginine, tweezers have been titrated to the free amino acids or short peptides, monitoring the Lys and Arg resonances [5,80]. While all resonances of the guest amino acid shift upon tweezer binding, the effect is most severe for the H atoms at the end of the side chain (H^ε^ for Lys and H^δ^ for Arg) since these are located right in the center of the aromatic tweezer cavity. In addition to the chemical shift perturbation, strong line broadening is observed, once again most pronounced for the H atoms located inside the tweezer cavity (Figure 2). Sulfonato-calix[4]arene has been titrated to trimethyl-lysine (KMe_3_), which yields the largest shifts and most severe line broadening for the methyl protons and H^ε^ resonances, indicating that these atoms are located inside the aromatic bowl of the calixarene [29]. The binding affinities of cucurbituril ligands for methylated lysine and arginine amino acids were determined using competitive ^1^H NMR titrations [49]. The supramolecular host is added to two competing guest molecules [85]. When the affinity for one of the guests is known, the affinity for the second guest can then be calculated from the relative amounts of the free guests and both complexes. This study revealed that the binding affinity of cucurbit[7]uril to methylated lysines increases with increasing number of methyl groups (KMe_3_ > KMe_2_ > KMe > K).

### Ligand-detected ^1^H NMR for compound screening

Ligand-detected ^1^H NMR methods have been well established for compound screening and fragment-based lead discovery (recently reviewed in [86–88]). In general, these methods can be divided into two categories according to their underlying principle. The first set of NMR-based screening methods relies on magnetization transfer via the nuclear Overhauser effect (NOE), e.g., saturation transfer difference (STD, reviewed in [89]), WaterLOGSY (recently reviewed in [90]) and transfer-NOE (trNOE, recently reviewed in [91]) experiments. The second set of methods is based on measuring the change of relaxation times (*T*_1_/*T*_2_/*T*_1ρ_) upon binding of a ligand to a protein. These methods require excess (5–20-fold) ligand together with relatively small protein concentrations (10–50 µM). Further advantages include no size limit for the protein, as well as no need for isotope labeling of the protein, assignment of protein resonances, and elaborate multidimensional NMR experiments.

While these methods can be useful for investigating supramolecular ligands binding to proteins, there are a few drawbacks one needs to keep in mind. All these methods require medium to weak (µM to mM) binding such that the binding kinetics lies in the intermediate to fast exchange regime because the ligand needs to dissociate again from the protein in order to be detected. Therefore, strong (nM or better) binders are not suitable for these experiments. No information about the binding site(s) on the protein is obtained. Magnetization transfer via NOE depends on a molecule’s correlation function and thus on its size. Small molecules <1000 g/mol show positive NOEs while large molecules like proteins exhibit negative NOEs. Around a molecular weight of ≈1000–1500 g/mol, the NOE passes through zero, meaning no NOE signal can be measured, even if molecules are in close enough proximity. A lot of supramolecular ligands fall in this size range and can thus appear as false-negatives. For methods like STD where resonances of the protein (usually methyl groups) are excited and magnetization transfer to the ligand is observed, the excited resonance must be far enough away from ligand resonances and this residue must also be in close enough proximity to the ligand binding site. In cases where supramolecular ligands contain peptidic scaffolds with methyl-bearing residues (like alanine, valine, leucine or isoleucine) whose NMR signals overlap with the protein methyl signals, finding a protein resonance that fulfills these prerequisites can be challenging. If a larger ligand binds to a smaller protein, signal overlap also becomes relevant for relaxation-based methods because the assumption that a protein signal relaxes a lot faster and thus does not contribute much to the overall signal intensity no longer holds true. While the transverse relaxation time *T*_2_ continuously decreases with increasing molecular weight, the longitudinal relaxation time *T*_1_ decreases with molecular size for small drug-like molecules, but increases with size for large molecules like proteins [92]. The minimum of the *T*_1_ vs. molecular weight curve also lies in the 1000–2000 g/mol range that many supramolecular ligands fall into. The *T*_1_ changes in this range are therefore ambiguous. As this review focusses on protein-based NMR techniques, we refer the reader to the cited review articles for more information on the ligand-detected techniques.

### ^1^H,^15^N-HSQC and ^1^H,^15^N-TROSY-HSQC spectra

^1^H,^15^N-HSQC NMR spectra are a widely used tool to study the interaction of proteins with natural or synthetic ligands as well as other biomolecules [83]. This spectrum requires ^15^N-labeling of the protein of interest, which is easily achieved by expression in minimal media in the presence of ^15^N ammonium salts. A ^1^H,^15^N-HSQC spectrum shows all N–H correlations that are moderately stable and do not exchange with the solvent water too quickly, which includes the amide NHs and some side chain NHs, e.g., the side chain amides of asparagine and glutamine as well as the indole NH of tryptophan and some of the arginine NH^ε^. This spectrum is also known as an amide finger print spectrum of a protein because the chemical shifts of the amides are very sensitive to both the secondary and tertiary structure. The amide signals, particularly for residues on the protein surface, are also sensitive to pH and in some cases ionic strength and type of counter ions. It is important to keep in mind that these factors can also affect the binding affinity of a ligand. The pH affects the protonation state, and thus charge, of both the protein and charged ligands as well, thus altering their electrostatic interactions. Around the isoelectric point, where a molecule is neutral, hydrophobic interactions become more pronounced, which can lead to formation of soluble aggregates or even precipitation.

Co-solvents such as DMSO-*d*_6_ are popular as solvents for more hydrophobic ligand stock solutions, but also can cause signal shifts in the NMR spectra. Therefore, a control titration with the co-solvent (but without ligand) must be performed and chemical shift perturbations need to be referenced to the spectrum with the same amount of co-solvent. Large amounts of co-solvent can induce unfolding of the protein, which is reflected by a collapse of signal dispersion in the HSQC spectrum, resulting in most amide signals moving into the random coil region between ≈7.5–8.5 ppm (in the ^1^H dimension). We found that a maximum of ≈5% of DMSO-*d*_6_ seems to be a good rule of thumb for many proteins, however the tolerance for co-solvents varies widely.

For larger proteins (>25–30 kDa), the transverse relaxation optimized spectroscopy (TROSY) versions of the ^1^H,^15^N-HSQC [93] can increase the signal-to-noise ratio of the NMR signals. Deuteration (C–D instead of C–H, partial or full) improves the relaxation properties of large proteins and thus helps to enhance the NMR signal. In addition, fast acquisition methods like the SOFAST-HMQC [94] or BEST experiments [95–96] can be used to record significantly more scans in the same time compared to classical HSQC experiments. In these experiments, the selective excitation of only the protons of interest, e.g., the amide HN, is achieved by using shaped pulses with an appropriate offset. The excitation bandwidth is chosen such that the solvent signal is not excited. Therefore, the spin-lattice relaxation rate (*R*_1_ = 1/*T*_1_) is enhanced because the energy can be more easily transferred to the solvent molecules without waiting for the bulk of them to relax. In turn, this allows a significantly shorter relaxation delay and thus a faster scan rate.

Upon addition of an unlabeled (and thus invisible) binding partner, a shifting of signals and/or decrease in relative intensity due to line broadening can be observed for amides in close vicinity of the binding site. Both the ^1^H and ^15^N chemical shifts of any given amide can change upon ligand binding, whereby the magnitude and direction of ^1^H and ^15^N shifts are independent.

The combined chemical shift perturbation is calculated from the geometric distance between the bound and free signals, including both ^1^H and ^15^N chemical shift differences Δδ_H_ and Δδ_N_ (Equation 1). Since the chemical shift range of ^15^N is ≈5-fold wider than for ^1^H, a weighing factor W_N_ for the ^15^N chemical shift is often applied. Since there is no one physically correct way to determine this weighing factor, different values have been used (for a more in-depth discussion see [83]). Based on the average variances for both nuclei observed for all amino acid residues in proteins listed in the BMRB data base, a ^15^N weighting factor of W_N_ = 0.154 was determined [97].

[1]
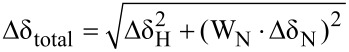


In the case of a two-state equilibrium, signals always shift in a straight line and produce a hyperbolic binding curve [83]. If the signal trajectory is curved, more than one binding site contributes to the chemical shift perturbation. In this case the resulting binding curves can be either hyperbolic, best fitting a model with *n* equal sites, or sigmoidal, which fits best to a cooperative binding model [98–99]. It needs to be kept in mind that the Hill coefficient obtained from the latter model is a measure of cooperativity and not automatically reflects binding stoichiometry [99]. Therefore, discrepancies to stoichiometries determined by other methods might occur. Furthermore, discrepancies can occur and fitting can be difficult if multiple binding sites with different binding constants are present, but the *K*_D_s differ not enough to yield clear biphasic binding. The commonly used binding models usually also do not account for potential ligand-induced formation of aggregates.

**Figure 3:**
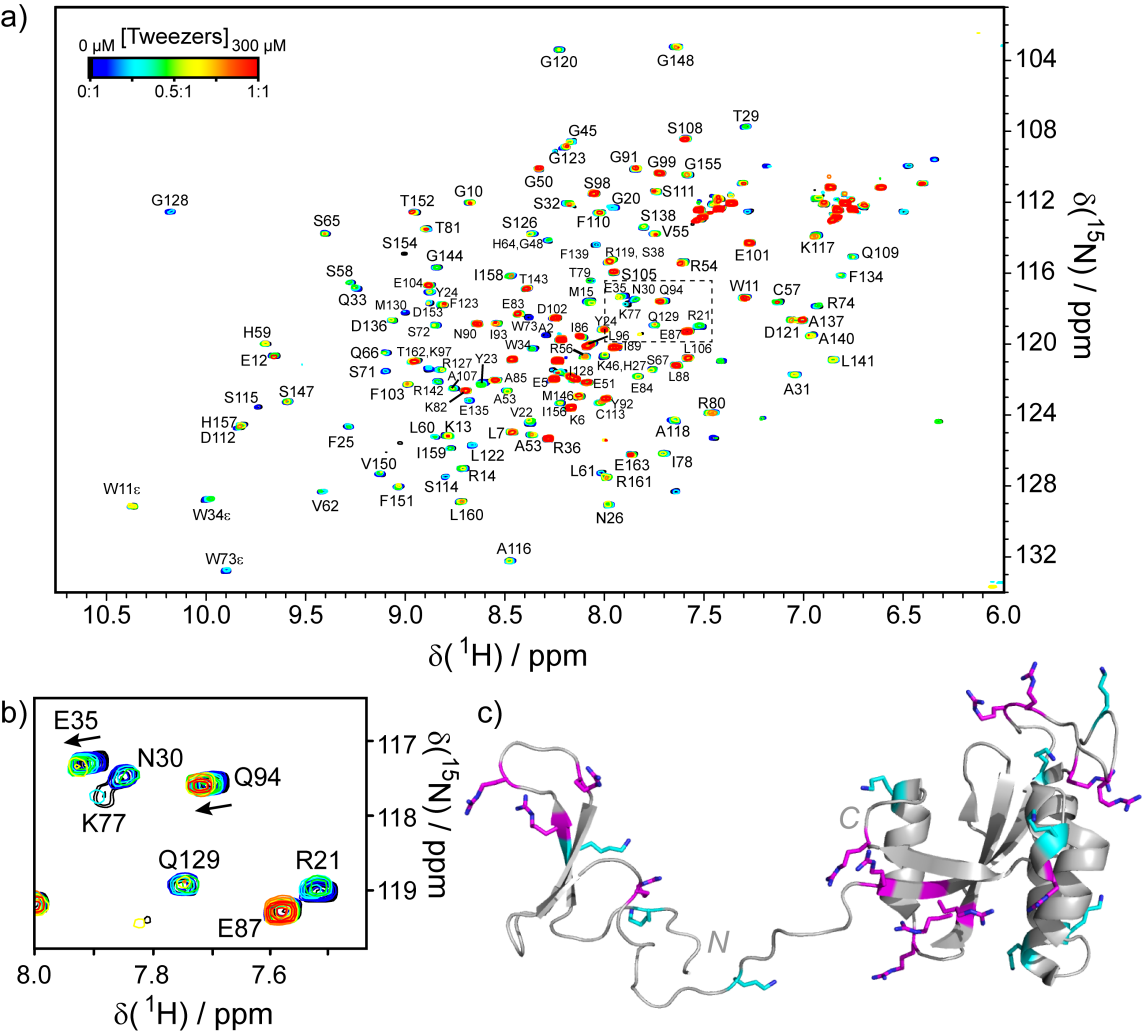
^1^H,^15^N-HSQC Titration of full-length *h*Pin1 with supramolecular tweezers (original data). (a) Spectra for different tweezer concentrations are overlaid and color-coded. Ligand binding causes a concentration-dependent shifting and/or broadening of the signals. At a 1:1 ratio of ligand to protein (red spectrum), many signals broaden. This can indicate the formation of soluble aggregates. (b) The expansion shows the area marked by the dashed box. Residue Q94 experiences a shifting of the signal upon tweezer binding (chemical shift perturbation). Residues R21, N30, K77 and Q129 show strong line broadening due to chemical exchange, which leads to a vanishing of the signals at low tweezer concentrations. Residue E35 shows both chemical shift perturbation and line broadening. Residue E87 is not affected by tweezers. (c) NMR Structure of *h*Pin1 (pdb 1NMV, [75]) with lysines highlighted in cyan and arginines highlighted in magenta.

For supramolecular ligands it is not uncommon that the sample starts to precipitate when excess ligand is added. The neutralization of surface charges upon binding of a charged ligand leads to a net reduction of charge in the complex, which likely increases the chance of aggregation due to hydrophobic interactions (see the red spectrum in Figure 3 as an example). Furthermore, some ligands exhibit a “molecular glue” effect by binding to two proteins simultaneously and thus induce aggregation [23–25,27,82]. This behavior does not interfere with identifying the ligand binding site, but it makes it difficult to determine a binding constant from the chemical shift perturbations since fitting a binding curve requires sufficient saturation of the signal. If a signal broadens beyond detection and thus disappears from the spectrum, be it due to aggregation or intermediate exchange kinetics, it is also impossible to trace its trajectory and determine the chemical shift perturbation.

In the case of a bifunctional GCP ligand binding to survivin [46], the ^1^H,^15^N-BEST-TROSY titration of a truncated survivin (residues 1–120) construct revealed one distinct interaction at the Asp and Glu-rich histone H3 binding site. This ligand was also able to inhibit the survivin-H3 interaction, which is important for cell division, in vitro and in vivo and reduce cancer cell growth.

Ruthenium(II) tris(bipyridine) (Ru^II^(bpy)_3_) complexes carrying multiple carboxylate-substituted arms were designed as protein surface mimetics, exploiting electrostatic binding through multiple contacts to the protein surface [63]. ^15^N-HSQC titrations showed that these complexes indeed recognize the highly positively charged area on cytochrome c (Cyt c) that binds Cyt c peroxidase. The inhibition of this protein–protein interaction was demonstrated as well using a luminescence quenching assay.

The protein surface recognition of two porphyrin ligands bearing carboxylate substituents binding to Cyt c was also studied by ^15^N-HSQC titrations [59]. Interestingly, large areas of the protein surface showed chemical shift perturbations, even though the binding curves clearly revealed a 1:1 stoichiometry. Crowley et al. concluded from the NMR data in combination with computational docking that the Cyt c/porphyrin complexes exist as a dynamic ensemble where multiple surface patches can transiently interact with the ligand through a combination of hydrophobic interactions with the porphyrin core and electrostatic interactions with the carboxylate substituents.

Cucurbit[7]uril and sulfonato-calix[4]arene binding to *Ralstonia solanacearum* lectin that was chemically dimethylated on three lysine residues and the N-terminus was studied by ^15^N-HSQC titrations [50–51]. Both ligands prefer binding to methylated lysines whereby the binding affinity (for the free amino acids) increases with the number of methyl groups [29,49]. On the protein surface, both ligands showed a clear preference for the sterically most accessible dimethyl-lysine residue K34Me_2_ which is located in a solvent-accessible loop. Binding of cucurbit[7]uril to this residue was confirmed by the co-crystal structure of the protein–ligand complex. In contrast, the native, unmethylated protein was not bound by cucurbit[7]uril.

The Hof lab developed sulfonato-calix[4]arene ligands with one or two additional aromatic substituents that show an even higher selectivity towards trimethyl-lysine over unmodified lysine than the unsubstituted parent ligand [31–32]. These ligands bind KMe_3_ in a short histone 3 (H3) peptide tight enough to even displace natural methyl-lysine binding proteins such as plant homeodomain (PHD) containing epigenetic readers. Since the H3 peptides are synthetic and not easily available in isotope-labeled form, a competition experiment was set up with ^15^N-labeled PHD domains. First, the methylated histone peptide was titrated to the PHD domain, resulting in chemical shift perturbations. The calixarene ligand was subsequently titrated to the same sample now containing the binary PHD–histone complex. Competition of the ligand for the peptide resulted in release of unbound PHD domain, which is evident from the signals shifting back towards the position of the free protein in the absence of peptide.

The Crowley lab has conducted multiple binding model studies comparing binding of sulfonato and phosphonato-calix[*n*]arenes of different ring sizes (*n* = 4, 6 or 8) to Cyt c [20,23–24,26–28] and the antifungal protein PAF [25] as model proteins. These studies combined ^15^N-HSQC titrations with X-ray crystallography, isothermal titration calorimetry (ITC) and size exclusion with light scattering (SEC-MALS), and have provided detailed insight into the binding mechanism of these supramolecular ligands. In general, the binding sites showing the largest perturbations in the NMR titrations largely agree with the sites observed in the crystal structures. Occasional discrepancies, where an additional site is observed by one method but not the other, are likely due to crystal packing effects. If crystal contacts between a ligand and additional copies of the protein are formed, this site might be favored in the crystal but not in solution. Conversely, a site that might be sampled in solution can be sterically disfavored in the crystal. The NMR titrations highlight large patches on the protein surface (up to 30–50% of the whole surface area) that cannot exclusively be explained by binding of the ligand to the crystallographic sites. These patches often contain multiple basic residues. Due to the moderate binding affinities, the calixarenes are thought to explore the protein surface dynamically, ‘hopping’ from one basic residue to an adjacent one. In addition, different binding modes involving the same residue have been observed in the crystals and can further contribute to this phenomenon. While steric accessibility is an important determining factor for calixarene binding, the overall local electrostatic potential of the protein surface as well as other non-covalent interactions such as hydrophobic interactions or aromatic ring stacking contribute to a certain selectivity for some lysine residues over others as well. The smallest calix[4]arene is confined to a bowl-shaped conformation and entraps mostly lysine side chains, however, occasionally an arginine residue is favored over lysine residues [80,82]. The larger calixarenes are more flexible, and different conformations have been found in the crystal structures, which enable a greater diversity of non-covalent interactions with the protein and even different binding modes on the same lysine side chain. With increasing ring size, the calixarenes promote ligand-induced ordered multimerization of proteins. Cyt c for example is monomeric in solution with the small calix[4]arene, while calix[6]arene induces protein dimerization [23], and calix[8]arene even tetramerization [24] in solution as evidenced by SEC-MALS. In the NMR spectra, the assembly of proteins is evident by increased line broadening of most residues due to the increase in particle size. Interestingly, a >2-fold excess of calix[8]arene over Cyt c results in a breaking up of the ligand-induced tetramers, which also results in a resharpening of the NMR signals at higher ligand concentrations [24]. This tendency to bridge protein molecules and assemble them into larger ordered structures makes especially the larger calixarenes potent “molecular glues” and crystallization aids [23–28]. The PAF protein for example did not yield diffracting crystals on its own, but good quality crystals were obtained in the presence of all calix[*n*]arenes [25]. The protein assembling properties of calixarene ligands can further be fine-tuned by introducing additional substituents [27–28].

Gordo et al. [40] designed a cationic guanidiniomethyl-calix[4]arene with two hydrophobic loops at the narrow rim to stabilize the tetramer interface of the p53 tetramerization domain (p53TD). ^15^N-HSQC titrations of the ligand showed sequential binding of the ligand at both edges of the tetramer interface for both the wild-type protein and its R337H mutant. Binding of the calixarene to p53TD-R337H resulted in a significant stabilization of the tetramer interface, which was severely destabilized by the mutation, while the stability of the wild-type tetramer was not affected.

The binding of several anionic supramolecular ligands to the model protein ubiquitin was compared using NMR by Crowley’s lab [82]. These ligands, including phosphate tweezers (CLR01), sulfonatocalix[4]arene (SCLX4), a sulfonated porphyrin derivative (TPPS), and pyrenetetrasulfonic acid (4PSA), all target the basic residues Lys and Arg on protein surfaces. Dependent on the shape and overall charge of the ligand, distinct preferences in binding sites could be deduced from the chemical shift perturbation and line broadening observed in the ^1^H,^15^N-HSQC titrations. The smallest ligand 4PSA showed the highest specificity, locating to the hydrophobic triad, where ubiquitin binds many of its protein interaction partners, which is surrounded by cationic residues. The flat, hydrophobic pyrene core can interact with the hydrophobic triad of the protein while the negative charges on the periphery of the ligand can interact with the surrounding positively charged amino acid side chains. A similar binding mode is likely for the also flat hydrophobic porphyrin. However, this TPPS ligand additionally binds another hydrophobic patch around residue I36 and the lysine-rich “belt” region. In contrast to the ligands with a flat scaffold, the bowl or ring-shaped calixarene and tweezers that encapsulate basic side chains prefer the arginine-rich C-terminus, as its residues are sterically more accessible.

We observed the binding of multiple tweezers to the same protein [7,80], which results in a shifting and broadening of many signals in the ^1^H,^15^N-HSQC, but the tweezers were not able to bind to all Lys and Arg residues on the protein surface. The titration of tweezers to the human peptidyl prolyl *cis-trans* isomerase *h*Pin1 (original data) is shown as an example in Figure 3.

On the regulatory N-domain of of the AAA+ ATPase p97 (p97-N), tweezers were found to bind only 6 patches even though this protein domain contains 30 Lys and Arg residues (26 of these are observable in the p97 crystal structure, pdb # 3CF3) [7]. The identified binding sites overlap with the region of p97-N that binds to the N-terminus of its cofactor UBXD1, and in fact, the tweezers were able to inhibit their interaction. Even upon tweezer binding to the small WW domain of *h*Pin1, one region between residues R17 to R21 is favored, even though a total of 6 basic residues are present [80]. Most of the identified patches bound by the tweezers contain two basic residues. QM/MM calculations [7] show that one of these residues is bound inside the tweezer cavity while the second residue can form a salt bridge with the second phosphate group of the tweezer. From the ^1^H,^15^N-HSQC data alone, it cannot be distinguished which one of these two residues is encapsulated in the tweezer cavity because the highly aromatic nature of the ligand causes chemical shift perturbations over a larger area on the protein surface due to its ring current effect. While the computational results suggest that a specific basic residue in a given patch is bound by the tweezers, a dynamic complex involving “ligand hopping” in solution cannot be excluded. This challenge can be solved with the Lys and Arg-specific side chain NMR spectra described below.

The binding of tweezers to the intrinsically disordered protein (IDP) tau was studied by ^1^H,^15^N-HSQC titrations using specific labeling of lysine residues [81], which will be described in the section ‘Specific amino acid labeling’.

**Figure 4:**
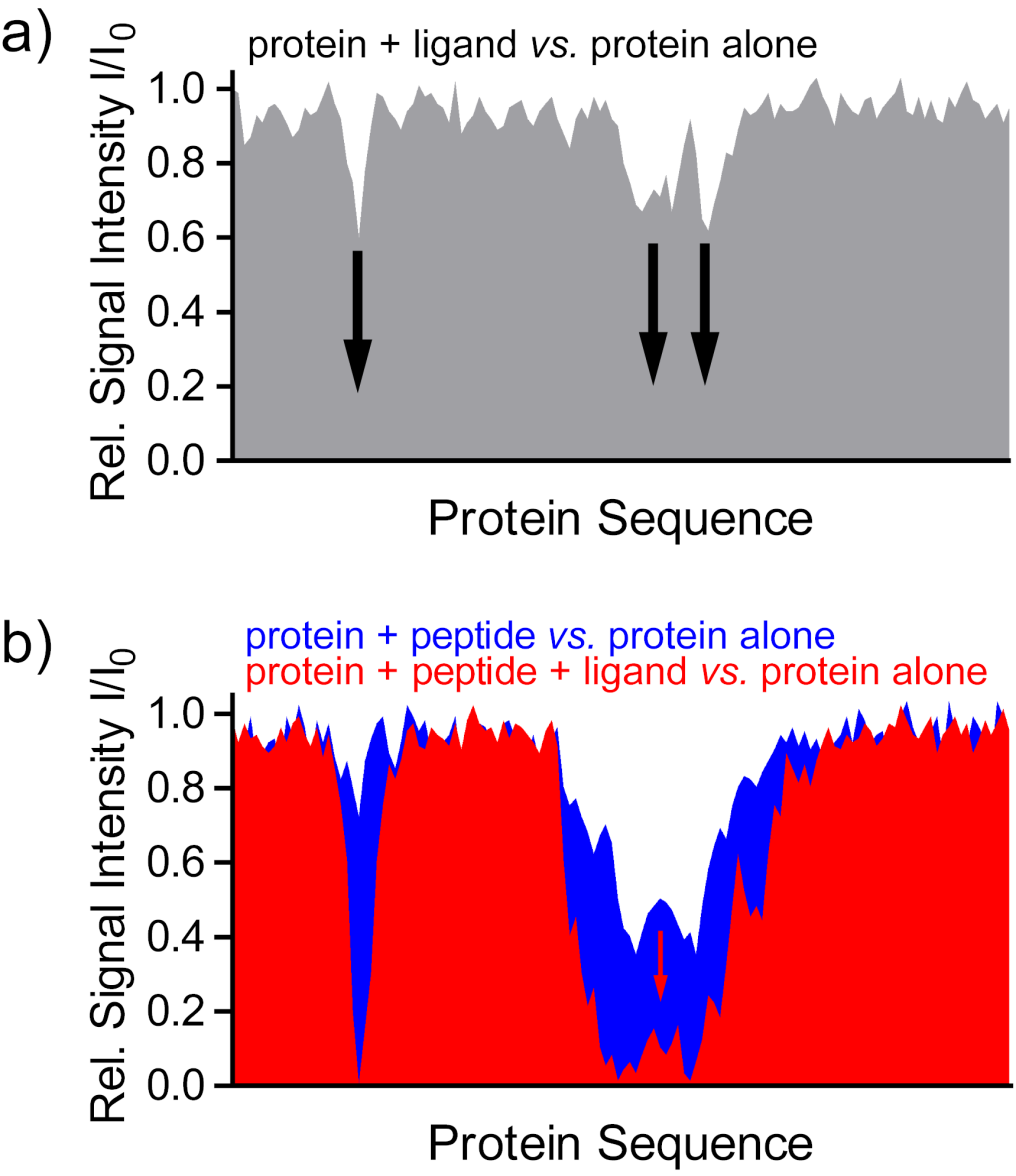
Relative signal intensities can be used to identify ligand binding sites (schematic representation of a hypothetical titration). (a) Binding of the ligand results in a reduction of the relative signal intensities *I*/*I*_0_ (protein with ligand vs. protein alone) of residues in proximity of the binding site due to line broadening. (b) The stabilizing effect of a ligand on a protein–peptide interaction in a ternary complex results in an additional decrease of relative signal intensities for the protein residues affected (red), compared to the binary complex of protein and peptide in the absence of a ligand (blue).

Multivalent GCP ligands have been successfully tailored to stabilize the interaction between the 14-3-3 protein and its phosphorylated cargo proteins [42,44–45]. Another ligand with just one GCP unit targets the central pore of the 14-3-3 dimer [43]. Unfortunately, these systems have not yet been studied by NMR. However, several other 14-3-3 inhibitors and stabilizers serve as great examples how the relative NMR signal intensities (bound form relative to free form of the observed protein) from ^1^H,^15^N-HSQC or TROSY-HSQC spectra can be used to identify the binding site and characterize the influence of a ligand on the protein–protein interaction. A hypothetical example to illustrate this principle is shown in Figure 4.

The following examples show that the methods presented are not limited to ligands binding to charged residues, but are generally applicable.^15^N-labeled phosphorylated tau protein was used as probe to monitor the binding of unlabeled 14-3-3 [100]. The relative signal intensities (tau bound by 14-3-3 compared to free tau) show a decrease for tau residues that are part of the 14-3-3 binding epitopes due to line broadening. In the presence of chimeric inhibitors, in which a small molecule was fused to a 14-3-3 binding peptide, the inhibitors target the unlabeled, and thus invisible, 14-3-3 and compete with tau binding. Therefore, binding of 14-3-3 to the ^15^N-labeled tau is less efficient, more free tau is present, and the decrease of tau signal intensities is less pronounced, approaching the intensities of free tau [101–102].

In recent studies, the reverse experiment, monitoring ^15^N,^2^H-labeled 14-3-3ΔC protein in ^1^H,^15^N-TROSY-HSQC experiments [103], was used to investigate the stabilizing effect of several compounds on the interaction of 14-3-3 with different unlabeled peptide epitopes derived from 14-3-3 cargo proteins [104–106]. E.g., a semi-synthetic ligand consisting of a fusicoccane diterpene core combined with different sugar moieties was designed to improve its properties as molecular glue for the 14-3-3/p53 complex [105]. Upon peptide binding in the absence of ligand, the 14-3-3 resonances of the binding site show reduced signal intensities. The relative intensities of the binary complex in the absence of a stabilizing ligand indicate kinetics of the binding equilibrium in the fast-to-intermediate time regime, resulting in line broadening. In the presence of a stabilizing ligand, a ternary complex consisting of 14-3-3, the peptide and the ligand is formed, resulting in a stabilization of the 14-3-3 peptide interaction. Due to this tighter binding, the dissociation of the peptide (*k*_off_) slows down. Therefore, the kinetics of the binding equilibrium shifts from fast-to-intermediate even more towards the intermediate time scale, which broadens the lines and decreases the signal intensities even more. In addition, the stabilization of the 14-3-3 peptide interaction in the presence of the ligand was confirmed by biochemical assays such as isothermal titration calorimetry (ITC) and fluorescence anisotropy.

On a quest for modulators of 14-3-3 protein–protein interactions, Valenti et al. used ^1^H,^15^N-TROSY-HSQC spectra of ^15^N,^2^H-labeled 14-3-3ΔC in combination with ligand-based WaterLOGSY experiments for the screening of a compound fragment library [107]. Screening by HSQC NMR spectra is more robust compared to ligand-detected NMR methods or fluorescence screening, yielding less false positive hits due to ligand aggregation or auto-fluorescence, while at the same time identifying the ligand binding site.

### Specific amino acid labeling

**Figure 5:**
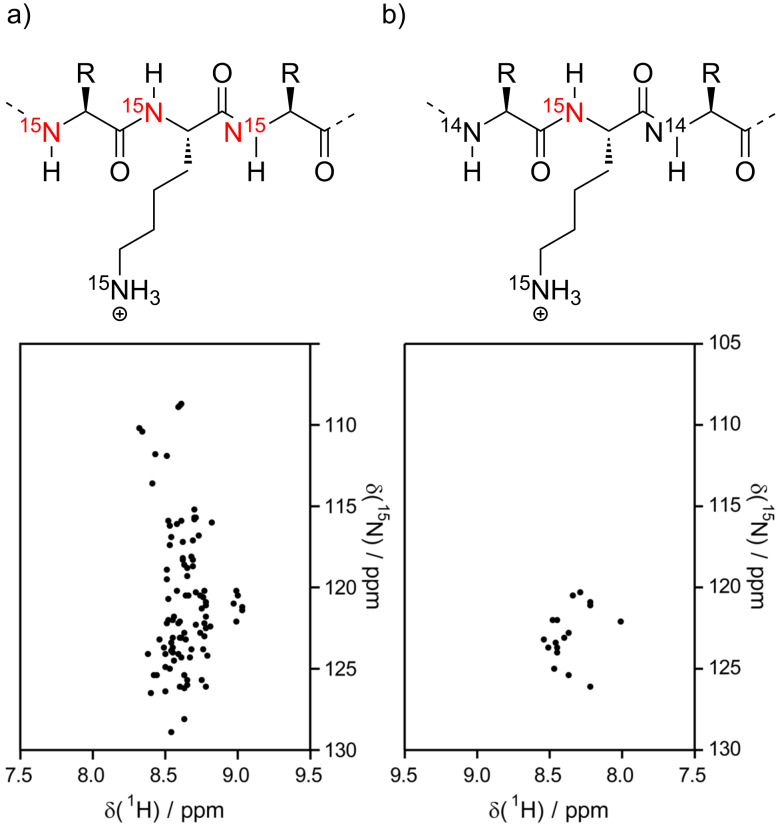
Schematic ^1^H,^15^N-HSQC spectrum of tauF4 (chemical shifts from BMRB # 17945, [109]) with and without specific ^15^N-lysine labeling. (a) Uniformly ^15^N-labeled protein. The amide NH of all residues (except Pro) yield a signal, resulting in signal overlap. (b) Selective ^15^N-lysine labeling. Only the amide NH of lysine residues are visible. The specific labeling significantly reduces signal overlap and thus makes it easier to track the shifting of single resonances upon ligand binding.

For larger and intrinsically disordered proteins (IDPs), signal overlap in ^1^H,^15^N-HSQC/TROSY-HSQC spectra becomes a challenge. This impedes not only the unambiguous assignment of all signals, but it also makes it difficult to gauge which one of the residues with signal overlap is responding to the binding of a ligand. For ligands targeting just one particular type of amino acids specific isotope labeling of just these amino acid residues is an elegant way to circumvent the aforementioned challenges. The supramolecular tweezers have been shown to inhibit amyloid fibril formation of the 441 residue full-length tau protein [81], which is one of the relevant culprits in Alzheimer’s disease and other tauopathies. Specific labeling of only Lys has been used to monitor tweezer binding to full-length tau with 44 lysines, showing that the tweezers can bind to multiple of them and thus interfering with tau aggregation. A schematic representation of a ^15^N-HSQC spectrum of a shorter tauF4 construct with uniform vs. lysine-specific labeling is shown in Figure 5. Lysine is particularly well-suited for specific labeling because it is not converted to other amino acids in the *E. coli* metabolism, which would lead to isotope scrambling and dilution. Therefore, specifically Lys-labeled proteins can easily be obtained by expressing the protein in unlabeled minimal media by simply adding the isotope-labeled amino acid, without the need for auxotrophic strains or metabolic enzyme inhibitors [108]. The assignment of all Lys residues, beyond the assignments available from classic uniform ^15^N- and ^13^C-labeling, was achieved by specific ^15^N,^13^C-labeling of Lys in combination with uniform ^13^C-labeling. A 3D-HN(CO)CACB experiment of this sample correlates the HN of each Lys residue with the ^13^C-labeled C^α^ and C^β^ atoms of the preceding residues, which enables determination of its amino acid type.

Selective ^15^N-arginine labeling has been described in [110] and was employed to protein–protein interactions and binding of small molecule inhibitors of the kelch-like ECH-associated protein 1 (Keap1) kelch domain. This labeling scheme will be interesting for supramolecular binders selective for arginine, but, to our knowledge, has not yet been applied in this context yet.

The specific labeling of glutamate is more challenging because this amino acid is easily metabolized into several other amino acid types. However, the specific incorporation of ^15^N-labeled glutamate in non-auxotrophic *E. coli* strains is possible using inhibitors for the metabolic enzymes converting glutamate [111]. The bacteria are grown in a modified M9 minimal medium containing the labeled amino acid, all others in unlabeled form, and the corresponding metabolic pathway inhibitors. To our knowledge, this method has not been applied to study the binding of ligands that recognize anionic residues on protein surfaces yet.

### ^1^H,^13^C-HSQC spectra

In principle, ^1^H,^13^C-HSQC spectra are also suitable to observe ligand binding to a protein that is isotope labeled with ^13^C. This spectrum shows all C–H correlations including the side chains CHs, which are usually in closer proximity to a binding ligand than the amides. The major disadvantage of this spectrum is the crowding of signals, especially in the region of the side chain CH_2_ groups, so tracking a single signal without running into signal overlap is challenging. However, side chain methyl groups show a larger signal dispersion and more favorable relaxation properties compared to CH_2_ groups, so they are mostly used to observe the interactions of a protein with its binding partners.

Especially methyl groups bound to a hetero atom like sulfur (in methionine) or nitrogen (e.g., in methylated lysine) have unique chemical shifts for both ^1^H and ^13^C nuclei. Methylation of Lys and Arg occurs as posttranslational modification (PTM) of proteins in nature, often used as a switch to regulate protein interactions and thus their function [112–113]. The Crowley lab has used this advantage to study the binding of sulfonatocalix[4]arene to dimethyl-lysine (KMe_2_) residues in the model protein lysozyme [22]. The preferred calixarene binding site was identified by tracking the signals of the lysine methyl groups, which can easily be identified in a ^1^H,^13^C-HSQC spectrum in the spectral region of 2–3 ppm for ^1^H and 40–45 ppm in the ^13^C dimension. The preferred, sterically most accessible, K116Me_2_ showed a large signal shift of > 0.5 ppm, while the secondary, less preferred site at K1Me_2_ showed significantly smaller signal shifts. The remaining four KMe_2_ residues did not show any signal shifts and thus no binding.

### Side-chain specific spectra (Lys/Arg)

While ^1^H,^15^N-HSQC titrations provide a very useful tool to map the binding area of any ligand on a protein surface, ligands targeting basic residues pose a challenge that make it difficult to unambiguously define which particular residue represents the preferred binding site. For several of these ligands, it has been shown that multiple lysines and arginines - but not all - on the same protein can be bound simultaneously. Furthermore, the aromatic nature of such ligands causes chemical shift perturbations over a larger area of the protein surface due to ring current effects. Especially the tweezers prefer patches with two adjacent positively charged residues, so that one of them is bound inside the tweezers’ cavity while the second one can form a salt bridge to the second tweezer phosphate group. With a ^1^H,^15^N-HSQC, that provides information for the amides only, it cannot be distinguished which one of these two residues constitutes the one encapsulated by the tweezer.

**Figure 6:**
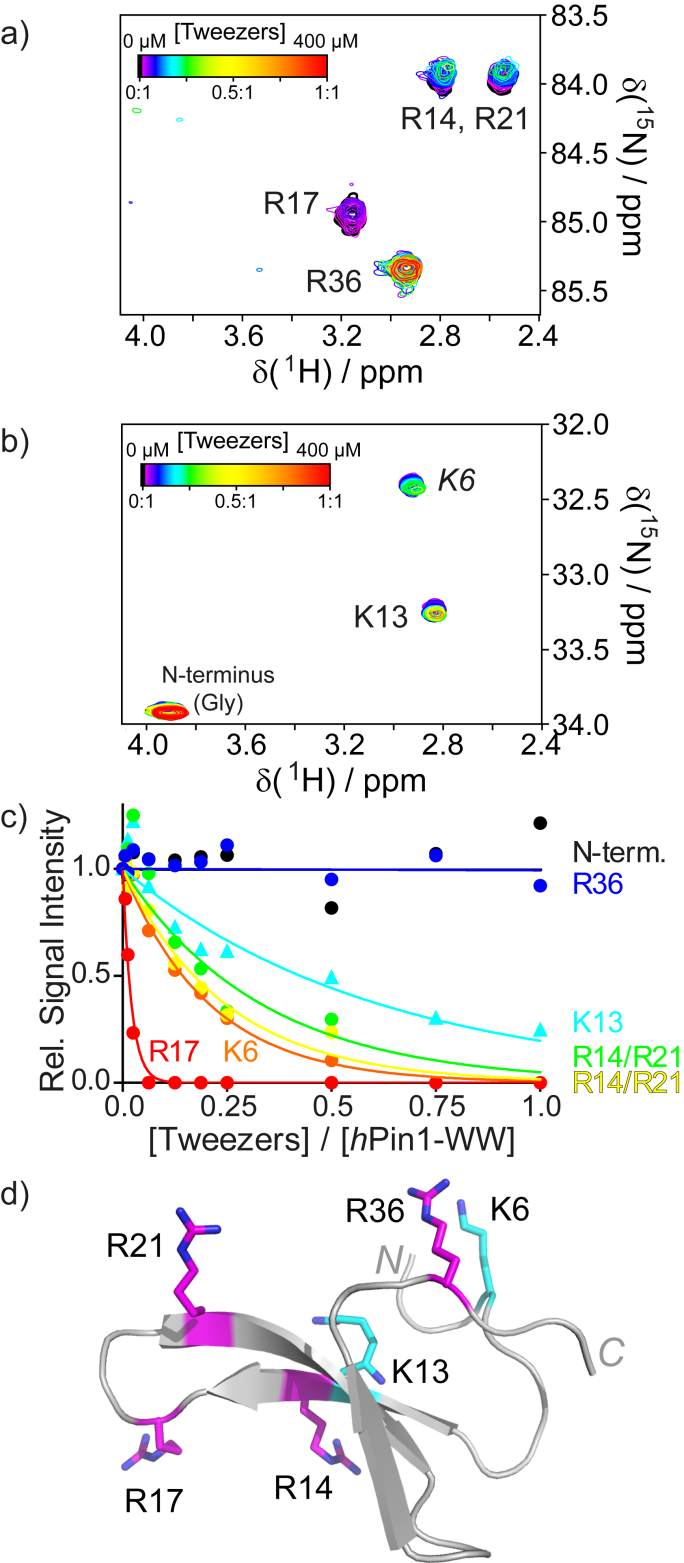
H2(C)N spectra specific for arginine (a) and lysine (b) residues of the *h*Pin1-WW domain at different tweezers concentrations (color-coded). The signals for R14 and R21 are both split and overlap. Upon tweezer binding, line broadening and thus reduced signal intensities are observed. (c) Plotting the relative signal intensities for each signal as a function of tweezers concentration (given in equivalents) reveals a distinct binding order. R17 is the preferred binding site, while R36 and the N-terminus are not bound at all. (d) Structure of the *h*Pin1-WW domain with lysines highlighted in cyan and arginines highlighted in magenta. Reprinted (reproduced) with permission from [80], copyright (2017) Wiley-VCH Verlag GmbH & Co. KGaA.

The side chain Hs, especially the ones at the end of the lysine or arginine side chain (H^δ^ for Arg, H^ε^ for Lys), are much better reporter nuclei because they are much more strongly affected by tweezer binding since they are located directly inside the aromatic cavity. Unfortunately, in the ^1^H,^13^C-HSQC, their signals appear in a very crowded region of the spectrum and tracking them is only possible for very small proteins [80]. We adapted Lys- and Arg-specific H2(C)N spectra first introduced by Iwahara [114–116] to circumvent this challenge [80]. These two-dimensional spectra require ^15^N and ^13^C-labeling of the protein and correlate the protons of the terminal CH_2_ group of Lys or Arg with the adjacent side chain nitrogen atom, passing the magnetization through their respective ^13^C nucleus (whose chemical shift is not recorded). Each one of these residues is represented by one signal in its respective spectrum (Figure 6). Lys- or Arg-selective (H2C)N(CC)H-TOCSY experiments are also available to aid in case the assignments for these residues are incomplete. The selection of Lys or Arg signals is achieved using shaped pulses that are specific for the respective side chain nitrogen atom (≈33 ppm for Lys N^ζ^ and ≈85 ppm for Arg N^ε^). N-terminal glycine residues are also visible in the Lys-specific spectrum because the chemical shifts of the C^α^H_2_-NH_3_^+^ moiety also match the selected chemical shift range. Not only is signal overlap reduced compared to the ^1^H,^13^C-HSQC because less signals appear in the spectrum, but also the observed signal dispersion is superior due to a larger dispersion in the ^15^N dimension. The H2(C)N spectra have proven to be very sensitive to tweezer and calixarene binding. Similar to the 1D-^1^H spectra of the single amino acids bound by tweezers, the signals from the terminal CH_2_ groups of the Lys and Arg side chains experience strong line broadening. The signals already lose intensity at very low ligand concentrations and disappear before significant signal shifting can be observed. Thus, the chemical shift dispersion cannot be used to determine binding constants. However, under the assumption that the binding equilibrium lies on the fast-to-intermediate time scale and that the on-rates for binding are similar for all residues, the signal decay as function of ligand concentration reflects the off-rate. A rapid signal decay indicates a slower off-rate and hence a tighter binding. While this approach does not yield absolute binding constants, it does allow a relative ranking of all possible ligand binding sites on the same protein. In contrast, classical biochemical methods for measuring binding affinities, such as isothermal titration calorimetry (ITC) or fluorescence anisotropy/polarization, are only able to provide an averaged *K*_D_.

## Outlook

In the last two decades, additional NMR methods have been developed to extend the size limits of NMR spectroscopy to very large multimers and heterogeneous multi-protein complexes by exploiting the favorable relaxation properties of methyl groups [117]. ^1^H,^13^C-Methyl-TROSY-HMQC experiments [118–119], recently reviewed in [120–121], in combination with selective ^1^H,^13^C labeling of methyl groups in otherwise ^2^H,^12^C-labeled proteins [122–123], extend the possibility to monitor binding events to very large protein assemblies by NMR. It has mostly been used to investigate protein–protein interactions within large multi-protein assemblies [120–121] and the effect of small natural ligands like ATP or ADP on the dynamics of multimeric proteins like the hexameric AAA+ ATPase p97 [124]. Furthermore, methyl-TROSY-HMQC has been employed to investigate the allosteric mode of action of the synthetic inhibitor filibuvir to the selectively Ile^δ1^ methyl-labeled hepatitis C virus RNA polymerase NS5B [125]. This study also includes a good description of the challenging assignment process, which entails single point mutations (all specifically isotope-labeled), distance measurements via NOE, and chemical shift predictions based on crystal structures. To our knowledge, methyl-TROSY has not yet been applied to monitor the binding of synthetic supramolecular binders to larger proteins or multimeric assemblies, but it could represent a useful tool in this context as well.

The assignment of the labeled methyl groups can be challenging and cost-intensive. Methionine scanning introduces single methionine (Met) mutants in a specifically ^13^C^1^H_3_-Met labeled protein [126] to circumvent this problem. Methionines have a naturally low abundance in proteins and their methyl resonances are easily identified. A methionine mutation, which is introduced around the suspected ligand binding site, is easily assigned because it adds one signal to the spectrum. This approach offers interesting options to study ligands targeting the surface or interfaces in larger proteins or protein assemblies.

## Conclusion

Protein-based solution NMR spectroscopy has long been the method of choice to map ligand binding sites at atomic resolution. With the recent improvements like fast acquisition techniques, selective isotope labeling of single or multiple types of amino acid residues or side chain specific spectra, NMR continues to thrive in the growing field of targeting protein surfaces with supramolecular chemistry. Even multiple binding sites on one protein surface can be identified and relative binding orders can be gauged. The combined analysis of chemical shift perturbations and changes in signal intensities makes NMR a suitable method as systems become more and more complex, such as multivalent and/or dynamic ligands, e.g., ultra-small nanoparticles coated with supramolecular recognition units, that are difficult to co-crystallize with proteins. We also see a future to expand the NMR toolbox to investigate supramolecular ligand binding to larger protein assemblies with methyl-TROSY in combination with specific methyl group labeling. Methionine scanning is an interesting option in this context because it circumvents the time and cost intensive need for a complete assignment of all methyl groups in the protein of interest.
